# Label-Free Quantitative Proteomic Analysis of the Global Response to Indole-3-Acetic Acid in Newly Isolated *Pseudomonas* sp. Strain LY1

**DOI:** 10.3389/fmicb.2021.694874

**Published:** 2021-08-10

**Authors:** Shuxue Zhao, Xi Chen, Qianshu Sun, Fei Wang, Chunhui Hu, Lizhong Guo, Jie Bai, Hao Yu

**Affiliations:** ^1^College of Environmental Science and Engineering, Ocean University of China, Qingdao, China; ^2^Shandong Province Key Laboratory of Applied Mycology, School of Life Sciences, Qingdao Agricultural University, Qingdao, China

**Keywords:** proteomics, microbial degradation, indole-3-acetic acid, *Pseudomonas* sp. LY1, stress response

## Abstract

Indole-3-acetic acid (IAA), known as a common plant hormone, is one of the most distributed indole derivatives in the environment, but the degradation mechanism and cellular response network to IAA degradation are still not very clear. The objective of this study was to elucidate the molecular mechanisms of IAA degradation at the protein level by a newly isolated strain *Pseudomonas* sp. LY1. Label-free quantitative proteomic analysis of strain LY1 cultivated with IAA or citrate/NH_4_Cl was applied. A total of 2,604 proteins were identified, and 227 proteins have differential abundances in the presence of IAA, including 97 highly abundant proteins and 130 less abundant proteins. Based on the proteomic analysis an IAA degrading (*iad*) gene cluster in strain LY1 containing IAA transformation genes (organized as *iadHABICDEFG*), genes of the β-ketoadipate pathway for catechol and protocatechuate degradation (*catBCA* and *pcaABCDEF*) were identified. The *iadA*, *iadB*, and *iadE*-disrupted mutants lost the ability to grow on IAA, which confirmed the role of the *iad* cluster in IAA degradation. Degradation intermediates were analyzed by HPLC, LC-MS, and GC-MS analysis. Proteomic analysis and identified products suggested that multiple degradation pathways existed in strain LY1. IAA was initially transformed to dioxindole-3-acetic acid, which was further transformed to isatin. Isatin was then transformed to isatinic acid or catechol. An in-depth data analysis suggested oxidative stress in strain LY1 during IAA degradation, and the abundance of a series of proteins was upregulated to respond to the stress, including reaction oxygen species (ROS) scavenging, protein repair, fatty acid synthesis, RNA protection, signal transduction, chemotaxis, and several membrane transporters. The findings firstly explained the adaptation mechanism of bacteria to IAA degradation.

## Introduction

Indole-3-acetic acid (IAA) is the main auxin in plants, which has been implicated in virtually all aspects of plant growth and development (Spaepen et al., 2007; Duca et al., 2014). IAA plays important roles in plant–microbe interactions as a signaling molecule and it is synthesized not only by plants but also by microorganisms, including bacteria and fungi (Spaepen et al., 2007; Duca et al., 2014; Arora et al., 2015; Fu et al., 2015; Xiao et al., 2017; Mehmood et al., 2019). IAA-producing microorganisms assist plant growth, including root elongation, colonization, and nitrogen fixation, and can effectively protect plants against various environmental stresses, such as salinity stress (Kang et al., 2019). On the other hand, IAA producers could manipulate IAA-related plant activities that can cause plant diseases (Lindow et al., 1998; Manulis and Barash, 2003). IAA-degrading strain, *Pseudomonas putida* 1290, could abolish the inhibitory effect to roots caused by exogenous IAA (Leveau and Lindow, 2005). It is suggested that IAA-producing bacteria and IAA-degrading bacteria play a role as IAA balancers, which affects the physiological processes of both plants and bacteria (Spaepen et al., 2007; Sun et al., 2018).

Microbial degradation of IAA has been studied for more than 60 years (Proctor, 1958; Kosuge et al., 1966; Egebo et al., 1991; Arora et al., 2015). Several IAA-degrading strains have been isolated and characterized (Proctor, 1958; Jensen et al., 1995; Leveau and Lindow, 2005; Ebenau-Jehle et al., 2012; Donoso et al., 2017; Greenhut et al., 2018; Lin et al., 2018). IAA aerobic degradation pathways in these strains have been studied, and these pathways could be classified as catechol pathways and anthranilate pathways. *Bradyrhizobium japonicum* and *Alcaligenes* strains have been reported to have the ability to catabolize IAA with anthranilic acid as the eventual degradation product. Two anthranilate pathways have been reported in *B. japonicum* strains. In one proposed pathway, IAA was metabolized via dioxindole-3-acetic acid, dioxindole, isatin, and isatinic acid to form anthranilate (Jensen et al., 1995). In the other postulated pathway, *B. japonicum* degraded IAA to anthranilate via 2-formamino-benzoyl-acetic acid and 2-amino-benzoyl-acetic acid (Egebo et al., 1991). *Alcaligenes* transformed IAA to anthranilate through isatin, and then anthranilate was further transformed to gentisate (Claus and Kutzner, 1983). Catechol is another end product of IAA degradation. IAA can be transformed to skatole via a decarboxylation reaction, and skatole can be further transformed to catechol via indoxyl, 2,3-dihydroxy-indole, and salicylic acid. Scott reported that *P. putida* 1290 could transform IAA through 2-hydroxy-indole-3-acetic acid, which is also a major degradation product of IAA in plants. 2-Hydroxy-IAA could be transformed into 3-hydroxy-2-oxo-IAA, which was further transformed to catechol (Leveau and Lindow, 2005; Scott et al., 2013). In these strains, catechol was presumably *ortho*-cleaved to *cis*,*cis*-muconate by catechol 1,2-dioxygenase (Leveau and Lindow, 2005; Scott et al., 2013; Greenhut et al., 2018).

The first gene cluster responsible for IAA degradation was reported in *P. putida* 1290. An 8,994-bp DNA fragment, containing the *iac* gene cluster *iacABCDEFGRHI*, in strain 1290 could confer to *P.* KT2440 the ability to grow at the expense of IAA (Leveau and Gerards, 2008; Lin et al., 2012; Scott et al., 2013; Greenhut et al., 2018). This *iac* gene cluster, which codes for the transformation of IAA to catechol, is not only distributed in strain 1290 but also in many other species. The IAA degradation capacity of at least six *iac* cluster-harboring strains has been confirmed (Leveau and Gerards. 2008; Greenhut et al., 2018). In strain *Enterobacter soli* LF7, an *iac* gene cluster, arranged as *iacHABICDEFG*, was identified (Greenhut et al., 2018). A catechol degradation cluster (*pcaIJD-catBCA*) is situated downstream of *iac* genes. The latter cluster was suggested to channel catechol into central metabolism through the β-ketoadipate pathway. The expression of the whole cluster, *iacHABICDEFG-mfs-pcaIJD-catBCA*, was controlled by a regulator IacR in strain LF7 and upregulated in the presence of IAA. *In vitro* experiments of *iac* genes provide more genetic evidence to microbial degradation of IAA. IacA proteins in *Acinetobacter baumannii* ATCC 19606 or *P. putida* 1290 have been proved to be able to transform IAA to 2-hydroxy-IAA and transform indole to indigo (Lin et al., 2012; Scott et al., 2013). The *iacE* and *iacC* gene products were tentatively proposed for the transformation of 2-hydroxy-IAA and dioxindole-3-acetic acid, respectively (Scott et al., 2013). Using the *Escherichia coli* cells harboring *iac* genes, Sadauskas et al. (2020) proved that IacE was responsible for the conversion of 2-oxoindole-3-acetic acid into 3-hydroxy-2-oxindole-3-acetic acid (DOAA). However, the biotransformation processes of IAA are still not very clear, and the involvement of other *iac* genes in the IAA degradation pathway remains more speculative.

Proteomics has been widely applied in the identification and quantification of proteins presented in a biological sample and provided a necessary foundation to generate insights into the complexities and molecular mechanisms under these complex systems (Armengaud, 2016; Kellie et al., 2019). These approaches have been used to investigate catabolic pathways and screen metabolic genes involved in these metabolic pathways (Li et al., 2016; Otzen et al., 2018; Lyratzakis et al., 2021). In addition, high-throughput proteomics analysis can be used to identify the global change in abundance of proteins due to adaptive responses of the microorganism using different substrates or under different environments. The bacterial adaptive responses to many pollutants or stress conditions have been deeply investigated (Li et al., 2012; Hartmann and Armengaud, 2014; Du et al., 2017; Liu et al., 2017; Xu et al., 2017; Chen et al., 2018); however, to date, the bacterial adaptive responses to indole or indole derivatives have not been reported.

In this study, a novel strain, *Pseudomonas* sp. LY1, was isolated from farm soil, which was able to use IAA as its sole source of carbon and nitrogen. The growth and degradation properties of strain LY1 were elucidated. Furthermore, we used label-free quantitative proteomics analysis to gain insights into the global adaptive response of strain LY1 to IAA degradation. Based on the genomic and proteomic analysis, a gene cluster responsible for IAA degradation was screened and the predicted genes in this cluster were comparatively analyzed. The main IAA degradation intermediates in strain LY1 were identified, and several genes in the gene cluster were silenced. Based on the genetic and chemical evidence, a proposed IAA degradation pathway in strain LY1 was presented.

## Materials and Methods

### Chemicals and Culture Media

Indole-3-acetic acid (IAA) and citric acid were purchased from Sangon Biotech (Shanghai, China). Methanol, formic acid, acetonitrile (ACN), indole, and isatin were purchased from Aladdin (Shanghai, China). Methoxyamine hydrochloride (MEOX), anthranilate, sodium dodecyl sulfate (SDS), ammonium bicarbonate, and urea were purchased from Sigma-Aldrich (Shanghai, China). *N,O*-Bis(trimethylsilyl)trifluoroacetamide (BSTFA) with 1% trimethylchlorosilane (TMCS) was purchased from Supelco (Shanghai, China). Trypsin was purchased from Promega Corporation (WI, United States). All other reagents used in this study were of liquid chromatography–mass spectrometry (LC-MS) or chromatographically grade and commercially available. The mineral salt medium (MSM) used in this study was prepared according to the previous description (Yu et al., 2018b). IAA and IAA analogs were added into MSM before autoclaving, and these compounds were stable after autoclaving. *E. coli* cells were cultivated in LB broth at 37°C as previously described (Yu et al., 2018b).

### Isolation and Identification of Strain LY1

The IAA-degrading strain was isolated from the farm soil in Qingdao (Shandong Province, China). Briefly, several soil samples were added into 50 ml sterilized MSM with 1,000 mg/L (5.71 mM) IAA and 1,000 mg/L yeast extract in a 250-ml flask for enrichment cultivation at 150 rpm 30°C. After 7 days of cultivation, 5 ml of the turbid culture was transferred into 50 ml sterilized MSM with 1,000 mg/L IAA and cultured under the same condition for selective cultivation. After 3 to 5 rounds of selective cultivation, the turbid culture was spread on MSM plate with 1,000 mg/L IAA and incubated at 30°C for 5 days. The dominant colony was selected for further analysis. The 16S rRNA gene of strain LY1 was amplified using primers 27F and 1492R with the genomic DNA of strain LY1 as the template according to the previous description (Zhao et al., 2019). Sequence alignment and phylogenetic analysis were performed by Mega 6.0 software (Tamura et al., 2013). The draft genome of strain LY1 was sequenced using Illumina Hi-Seq 2500 system (CA, United States) and assembled with Velvet software as previously described (Zerbino and Birney, 2008; Yu et al., 2011).

### Growth and IAA Degradation of Strain LY1

Strain LY1 was cultivated at various pH (7.0–9.0) and different temperatures in the range from 23 to 37°C to assess the effect of pH and temperature on cell growth, respectively. The cell growth and IAA degradation of strain LY1 with different concentrations of IAA were measured under optimal conditions. IAA concentration was determined using high-performance liquid chromatography (HPLC) (Jensen et al., 1995). The growth of culture was measured according to the absorbance at 600 nm (OD600). Resting cell reactions were performed to characterize the transformation of IAA by strain LY1. Cells cultivated with IAA or citrate/NH_4_Cl, respectively, in the late exponential phase were collected by centrifugation at 8,000 × *g* for 5 min at room temperature. The cell pellet was washed twice with 50 mM PBS (pH 7.5) and re-suspended in the same buffer to OD600 = 6.0, which was designated as resting cells. After adding IAA into the resting cells, samples were withdrawn at regular interval times and centrifuged at 10,000 × *g* for 2 min. The supernatant was transferred to a new tube and stored at −20°C for further analysis. All experiments were repeated at least three times, and statistical analysis was performed using the two-sided *t*-test.

### Protein Extraction and Enzymolysis

Strain LY1 was grown in IAA or citrate/NH_4_Cl as the sole source of carbon and nitrogen, respectively. Cells in the late exponential phase (OD600 = 0.6 ∼ 0.8) were collected by centrifugation (8,000 × *g*, 5 min, 4°C), and washed twice with PBS (pH 7.5). Then, cells were re-suspended with 1 ml extraction buffer (2% SDS, 100 mM Tris–HCl, 1 mM EDTA, 1 mM PMSF, pH 7.6) and lysed with QIAGEN TissueLyser II (Hilden, Germany) at 150 Hz for 60 s. Cell debris was removed by centrifugation at 12,000 × *g* for 10 min at 4°C, and the supernatant was transferred to a new tube. The protein concentration was measured with microBCA Protein Assay Kit (Thermo Fisher, CA, United States) before adding DTT. Enzymolysis was performed with FASP methods according to the methods of Wiśniewski et al. (2009).

### LC-MS/MS Data Analysis

Desalted peptides were reconstituted in 10 μl 0.1% formic acid. The peptides were analyzed using a Nano-LC system coupled with Orbitrap Fusion Tribrid (Thermo Fisher, CA, United States). The MS instrument was operated in data-dependent acquisition mode, with full MS scans over a mass range of *m*/*z* 350–1,500 with detection in the Orbitrap (120 K resolution) and with auto gain control set to 100,000. Different chromatographic gradient lengths from 60 to 240 min were tested for peptide separation. All gradient started at 5% (v/v) ACN (0.1% formic acid) and went up to 32% (v/v) ACN (0.1% formic acid). Peptide identification and quantitation were performed by the Proteome Discoverer software suite (v2.0; Thermo Fisher Scientific, CA, United States) and the Mascot search engine (v2.4; Matrix Science, CA, United States). The data were searched against the protein database of strain LY1. The data of one group comes from four samples, two biological replicates and two technical replicates for each biological sample. The two-sided *t*-test was performed to detect fold change between each protein; *p*-value < 0.05 was considered significant.

### RT-qPCR

Cells cultivated in the presence or absence of IAA were used for RNA preparation. Total RNA and cDNA were prepared according to the previous description (Yu et al., 2018a). Primers used for RT-qPCR analysis are listed in Supplementary Table 1. RT-qPCR reactions were performed in a volume of 20 μl on an ABI QuantStudio 5 (Thermo Fisher Scientific, CA, United States) with ChamQ Universal SYBR qPCR Master Mix (Vazyme, Nanjing, China) according to the manufacturer’s instruction. The threshold cycle (*C*_*T*_) values were normalized to the expression level of the 16S rRNA gene. Expression levels were calculated according to the 2^–^*^Δ Δ CT^* method.

### Mutation Construction of the Candidate Genes Responsible for IAA Degradation

Partial DNA fragments of *iadA*, *iadB*, and *iadE* genes were removed from the genome of strain LY1, respectively, using a homologous recombination gene replacement system as previously described (Yu et al., 2018a). Briefly, two fragments of *iadA*, *iadB*, or *iadE* were amplified and ligated into pK18mobsacBtet (primers designed for this study are listed in Supplementary Table 1), and then the double-crossover recombinants (*Pseudomonas* sp. LY1Δ*iadA*, *Pseudomonas* sp. LY1Δ*iadB*, and *Pseudomonas* sp. LY1Δ*iadE*) were screened as previously described (Yu et al., 2018a).

### Ultraviolet (UV) Spectroscopy, HPLC, LC-MS, and NMR Analysis

Ultraviolet (UV) spectrum scanning was performed on an Agilent Cary60 spectrophotometer. HPLC was performed on Waters Alliance equipped with diode array detector using reverse-phase column (Waters Spherisorb ODS2, 4.6 mm × 250 mm, 5 μm, Massachusetts, United States) at 30°C. The mobile phase contained 35% (v/v) methanol and 65% distilled water (containing 0.05% formic acid) at a flow rate of 1.0 ml/min. LC-MS analysis was performed on a Thermo Fisher Orbitrap Fusion; Tribrid equipped with electrospray ionization (ESI) sources using a reverse-phase C18 column (Agilent ZORBAX RRHD Eclipse Plus C18, 2.1 mm × 100 mm, 1.8 μm, CA, United States). The mobile phase was 30% (v/v) methanol and 70% distilled water (containing 0.05% formic acid) at a flow rate of 0.2 ml/min. Positive and negative electrospray ionization analysis with the continuous full scanning from *m*/*z* 50–500 were collected. Samples for HPLC or LC-MS analysis were treated by centrifugation at 10,000 × *g* for 5 min and the supernatant was filtered by the 0.22-μm-pore-size filter. The degradation intermediates were purified by collecting the peaks in HPLC analysis. For chromatography–mass spectrometry (GC-MS) analysis, the samples were dried by nitrogen flushing and dissolved in 50 μl of 25 mg/ml MEOX dissolved in pyridine. The mixture was incubated for 30 min at 60°C. Then 50 μl BATFA + TMCS was added to the sample and the mixture was vortexed. The derivatization was performed by standing the reaction mixture for 90 min at 30°C. Samples were maintained in dark at 4°C until GC-MS analysis. GC-MS analysis was performed on Thermo Fisher TSQ 8000 EVO triple quadrupole mass spectrometer coupled with a Trace 1300 gas chromatograph (Thermo Fisher Scientific, CA, United States).

### Nucleotide Sequence Accession Numbers

The 16S ribosomal RNA gene sequence of *Pseudomonas* sp. LY1 is available in GenBank under accession number MK860766. The genome sequence of *Pseudomonas* sp. LY1 has been deposited at DDBJ/ENA/GenBank under the accession LSSW00000000. The version described in this paper is version LSSW01000000. This strain can be obtained from China Center for Type Culture Collection under the accession number of CCTCC AB 2016308. Proteome data are available via ProteomeXchange with identifier PXD014968 (Reviewer account: Username: reviewer42287@ebi.ac.uk, Password: aHDIThrQ).

## Results

### Isolation and Characterization of Strain LY1

Strains that could use IAA as the sole source of carbon and nitrogen were isolated from farm soil in Qingdao, and the dominant colony was selected from the MSM agar plate with IAA as growth substrate by serial dilution. The strain was designated as LY1. A phylogenetic analysis of the 16S rRNA gene sequence of strain LY1 with those of other members of the genus *Pseudomonas* was performed (Supplementary Figure 1). The 16S rRNA gene of strain LY1 showed the highest sequence identity (98.7%) with that of *Pseudomonas composti* C2. To obtain the optimum condition under which *Pseudomonas* sp. strain LY1 can most efficiently break down IAA, strain LY1 was cultivated with different pH and temperatures in MSM containing 1,000 mg/L IAA. As presented in Supplementary Figures 2A,B, the optimal pH for the growth of strain LY1 is 8.0. The optimal growth temperature of strain LY1 is 30°C, and there is no significant difference between 30 and 37°C. Supplementary Figure 2C shows the growth of strain LY1 with different concentrations (in the range of 500-3,000 mg/L) of IAA. Strain LY1 could grow on IAA at an initial concentration up to 3,000 mg/L. The highest cell density increased with the increase of the IAA concentration. High IAA concentration (2,000–3,000 mg/L) slightly affected the length of lag-phase of strain LY1. IAA could be completely transformed at all concentrations. After 33 h of cultivation, the IAA removal rate was ∼98% with 3,000 mg/L initial concentration of IAA (Supplementary Figure 2D).

### IAA Degradation in Strain LY1

**FIGURE 1:**
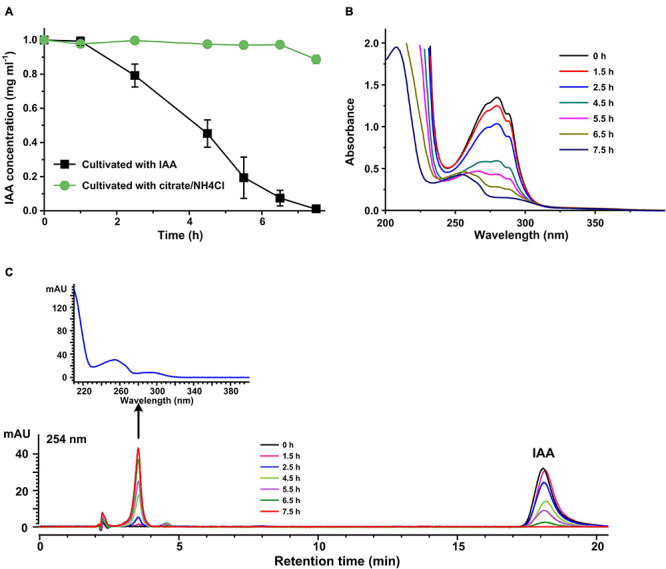
Indole-3-acetic acid (IAA) transformation by resting cells of strain LY1. **(A)** IAA (2,000 mg/L) degradation by resting cells of strain LY1 cultivated with IAA (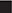) and citrate/NH_4_Cl (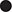), respectively. The cell density of resting cells is OD600 = 6.0. The reaction was performed at 30°C 120 rpm. **(B)** UV scanning of the samples at interval times from IAA transformation reactions. The reactions were performed with resting cells of strain LY1 cultivated with IAA. **(C)** HPLC analysis of the samples at interval times from IAA transformation reactions. The reactions were performed with resting cells of strain LY1 cultivated with IAA. The signal at 254 nm was recorded. The arrow indicated the UV spectrum of the compound with a retention time of 3.60 min. The mobile phase was 35% (v/v) methanol and 65% (v/v) 0.05% formic acid. The HPLC column was Agilent XBD C18 (4.6 mm × 250 mm, 5 μm). Each value is the mean from three parallel replicates ± SD.

Indole-3-acetic acid transformation reactions were performed by resting cells of strain LY1 prepared from MSM + IAA or MSM + citrate/NH_4_Cl, respectively. IAA was transformed rapidly by IAA-grown LY1 cells, and the IAA was almost all transformed within 7.5 h. IAA concentration in resting cells reaction with non-IAA-grown cells was basically unchanged (Figure 1A). The results indicated that the expression of enzymes responsible for IAA transformation in strain LY1 was induced by IAA. The UV scanning result of samples from the resting cell reactions by IAA-grown cells is presented in Figure 1B. The spectra representing IAA gradually declined with time, and the shape of the spectra gradually changed with time, indicating that IAA was transformed into a new compound(s). HPLC analysis of these resting cells reactions samples is shown in Figure 1C.

### Genomic Sequencing and Proteomic Quantification

To profile the protein abundance induced by IAA in strain LY1, genome sequencing and quantitative proteomic analysis based on the label-free method was executed. Genome sequencing was performed by Illumina Hi-Seq 2500 sequencer with reads of 150-bp length. The reads were assembled to 34 large contigs (>1,000 bp) using Velvet software. The N50 was approximately 27.6 kb. The genome sequence is 5,265,005 bp in length with a G + C content of 62.99%. Gene prediction and genome annotation were carried out using the RAST auto annotation server (Overbeek et al., 2014). The genome encodes 4,805 putative coding sequences (CDSs).

**FIGURE 2:**
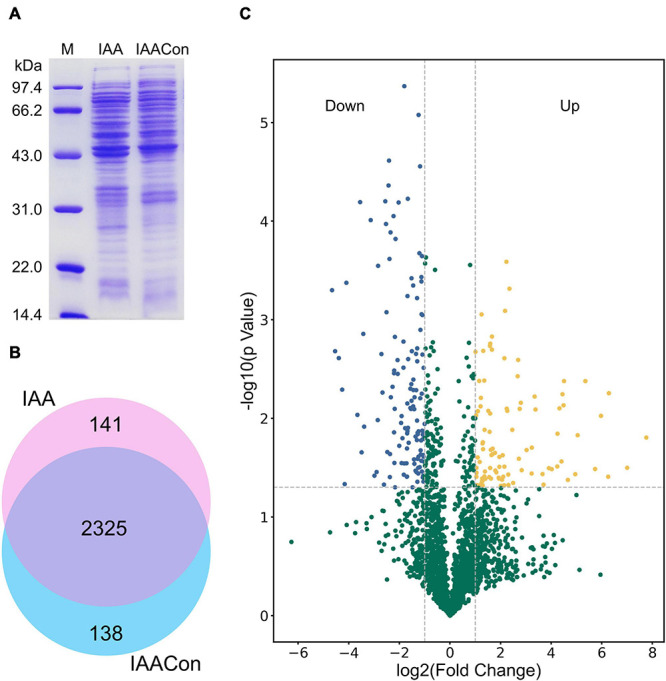
SDS-PAGE, Venn diagram, and volcano plot of the proteomic dataset. **(A)** SDS-PAGE analysis of crude proteins extracted from cell of IAA group (cultivated with IAA) and IAACon group (cultivated with citrate/NH_4_Cl). **(B)** Venn diagram of proteins identified in the proteomes of IAA and IAACon group. **(C)** Volcano plot showing changes in protein abundance from IAA and IAACon group.

**FIGURE 3:**
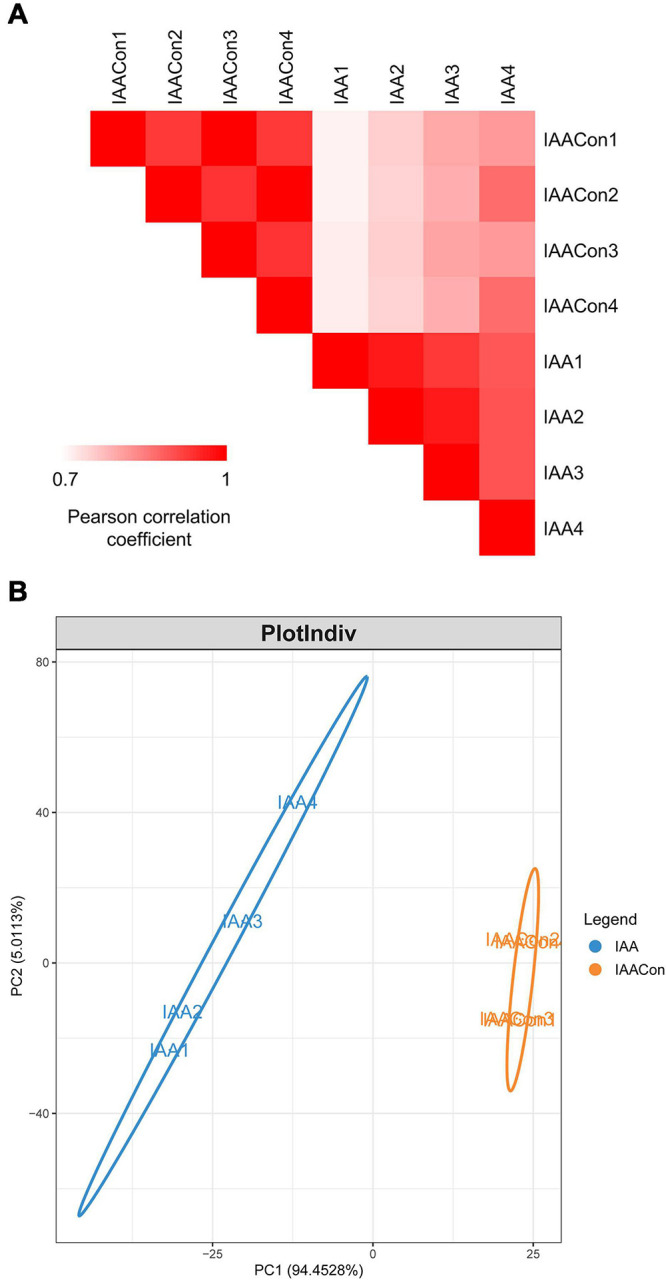
Overview of proteome data. **(A)** Pearson correlation coefficients for pair-wise comparisons of the IAA and IAACon proteome data. **(B)** PLS-DA of proteome data from IAA and IAACon samples.

Protein samples of strain LY1 cultivated in MSM with IAA (IAA group) and MSM with citrate/NH_4_Cl (IAACon group), respectively, in two independent experiments were prepared (Figure 2A). A total of 2,604 proteins were identified after filtration in 8 runs of protein samples (Supplementary Table 2). A 2.0-fold change cut-off and *p*-value < 0.05 were used to categorize proteins with differential abundances (DAPs). Compared with the IAACon group, the IAA group was associated with 97 proteins that were upregulated and 141 proteins were unique to the IAA group (Figures 2B,C). These 238 proteins were designated as IAA-up DAPs. The abundance of 130 proteins was downregulated in the IAA group compared with those from the IAACon group and 138 proteins were unique to IAACon group (Figures 2B,C). These 268 proteins were designated as IAA-down DAPs. Significant differential abundance profiles were observed when the Pearson correlation coefficients between the IAA group and the IAACon group were calculated. Moreover, IAA had more divergent abundance levels than IAACon (Figure 3A). In addition, we performed partial least squares-discriminant analysis (PLS-DA) and established that samples from the IAA group and the IAACon group could be divided into two groups by the first component (Figure 3B). These results revealed that the proteome data could represent the differences between the two groups.

### Functional Categorization Analysis

**FIGURE 4:**
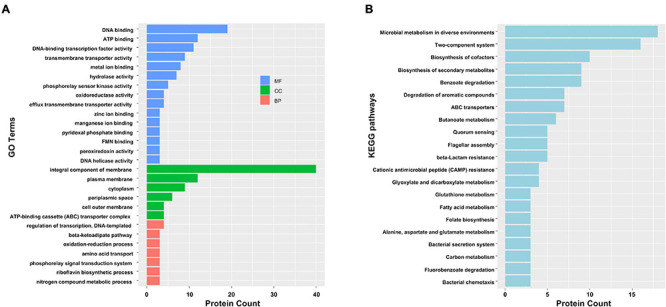
Functional categorization analysis of upregulated DAPs in the presence of IAA. **(A)** Major enriched GO terms of IAA-up DAPs in molecular function (MF), cellular component (CC), and biological process (BP) terms. The statistics with more than 3 proteins at GO level 2 are shown in this figure. **(B)** Major enriched KEGG pathways of IAA-up DAPs with at least three DAPs.

To gain functional information about DAPs, GO annotations enrichment and KEGG pathways enrichment of IAA-up DAPs were performed (Figure 4). Under the category of molecular function, IAA-up DAPs were mainly found in DNA binding, ATP binding, transmembrane transporter activity, metal ion binding, and hydrolases. In the cellular components, IAA-up DAPs were mainly enriched in membrane, cytoplasm, and ATP-binding cassette transporter complex. In the biological process, IAA-up DAPs were mostly related to the regulation of transcription, beta-ketoadipate pathway, and oxidation–reduction process (Figure 4A). The IAA-up DAPs were associated with 66 specific KEGG pathways, which were mainly distributed in microbial metabolism in diverse environments (path: 01120), two-component system (path: 02020), biosynthesis of cofactors (path: 01240), benzoate degradation (path: 00362), biosynthesis of secondary metabolites (path: 01110), degradation of aromatic compounds (path: 01220), ABC transporters (path: 02010), butanoate metabolism (path: 00650), beta-lactam resistance (path: 01501), flagellar assembly (path: 02040), and quorum sensing (path: 02024) (Figures 4A,B).

**FIGURE 5:**
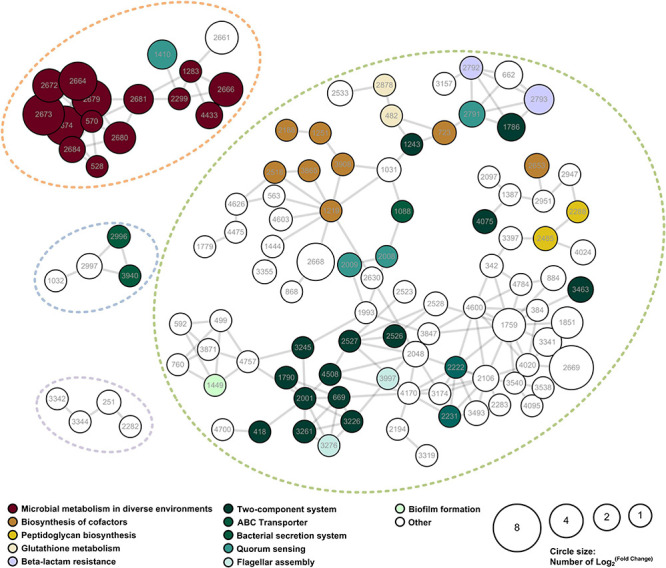
PPI network of the IAA-up DAPs were searched for their PPI using the web resource STRING and characterized using Cytoscape. Networks with at least 3 elements are shown in the figure. The size of the circle denoted the fold-change of the protein. Fold-change of the DAPs that were unique to the IAA group was set as 2. The enriched KEGG functions of the DAPs are indicated in different colors.

A PPI network of the IAA-up DAPs was constructed to reveal how the DAPs are related to multiple interaction pathways. Figure 5 exhibited that most IAA-up DAPs (183 proteins) had direct interactions with each other. DAPs were grouped into four networks that contained at least four proteins (Figure 5). Network 1 contains 16 proteins mainly associated with microbial metabolism in diverse environments. These proteins were considered to be directly related to IAA degradation. Most proteins (159) were grouped in network 2 and mainly associated with cofactors synthesis, transportation, and quorum sensing processes. The upregulation of proteins in networks 2, 3, and 4 were induced by strain LY1 to adapt to IAA degradation.

Functional enrichment and PPI network analysis suggested that several proteins related to stress response were significantly upregulated. These proteins include universal stress protein, catalases, superoxide dismutases, glutathione metabolism proteins, cold shock proteins, and chaperons (Supplementary Table 3).

### Investigation of Molecular Mechanism Under IAA Degradation in *Pseudomonas* sp. LY1

#### Screening Gene Cluster Responsible for IAA Degradation

Genes responsible for pollutant degradation in bacteria are usually specifically expressed and clustered in the genome. Therefore, to screen genes responsible for IAA degradation, gene clusters with 3 genes in 10 continuous genes in the genome or 5 genes in 15 continuous genes in genome are listed in Supplementary Table 4 as candidate gene clusters. The average fold-change level (AFCL) of each gene cluster was calculated (Supplementary Table 4) and the clusters were ordered by AFCL values. According to previous studies, gene cluster with the highest AFCL and with at least five genes were most likely to be the gene cluster responsible for pollutant degradation. Based on this criteria, gene cluster 1 was selected which has a much higher AFCL value (40.89) than that of cluster 2 (8.24).

**FIGURE 6:**
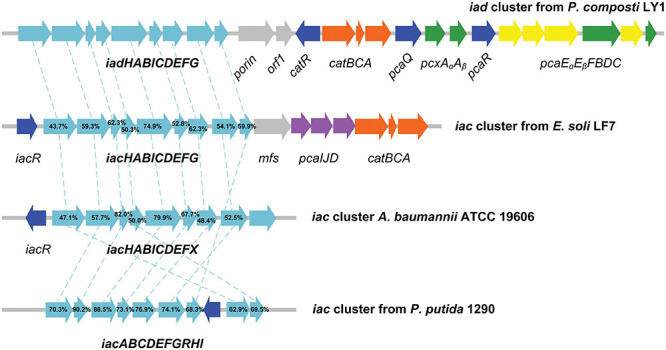
Graphical representation of *iad* gene cluster in strain *Pseudomonas* sp. LY1. Arrows indicate the size and direction of transcription of each gene. The numbers above the arrows indicate the amino acid sequence identity between the marked protein and the corresponding orthologous protein from *Pseudomonas* sp. LY1. Highlighted in cyan are the *iad* (*iac*) genes, in blue are the regulator genes, in orange are the *catBCA* genes, in purple are the *paaIJD* genes, in gray are the genes with unknown function, and in yellow and green are the *pcaABCDEF* genes.

Cluster 1, designated as IAA degrading gene cluster (*iad* cluster), has three predicted transcription units which are separated by two regulator coding genes (Figure 6). The functions of genes in the *iad* cluster were predicted by sequence alignment. As we can see from Figure 6, the organization of *iad* genes (*iadHABICDEFG*) is similar to that in strain *E. soli* LF7 (Greenhut et al., 2018) and *A. baumannii* ATCC 19606 (Shu et al., 2015) but different from that in strain *P. putida 1290* (*iacABCDEFGRHI*) (Scott et al., 2013). No regulator was predicted in the upstream of *iad* genes; instead, a regulator gene, *iadR1*, is located downstream of *iad* genes after a porin coding gene and a hypothetical protein-coding gene (*orf1*). The downstream genes of *iadR1* include *catBCA* (responsible for the conversion of catechol to β-ketoadipate enol-lactone) and *iadR2* → *pcaA_α_ A_β_* → *iadR3* → *pcaE_α_ E_β_ FBDC* (coding for the conversion of protocatechuate to succinyl-CoA and acetyl-CoA through β-ketoadipate enol-lactone pathway).

**FIGURE 7:**
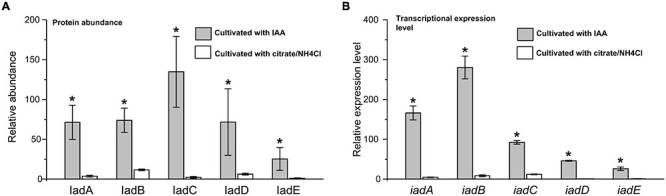
Expression level of IAA degrading relative genes in *iad* cluster. **(A)** Protein abundance levels of IAA degrading enzymes in the IAA group (gray bar) and the IAACon group (white bar). **(B)** qPCR analysis of the IAA degrading genes that were differentially expressed in IAA group (gray bar) and IAACon group (white bar). The least protein abundance and expression level of *iadE* in the IAACon group were set as 1. *t*-test was used to calculate statistical significance between IAA and IAACon group. *p < 0.05.

Proteomic analysis indicated that almost all the detectable proteins in the *iad* gene cluster were significantly upregulated in the presence of IAA compared with those without IAA. The IadHABICDEFG proteins were upregulated with ≥4.0 ratios. CatA and CatC proteins were upregulated with ratios ≥ 15. Pca proteins were also upregulated with more than 4-fold (except for PcaB of 1.5-fold). To confirm the protein differences at the transcript level, the mRNA expression of five differentially expressed key genes were analyzed. The results showed that mRNA expression levels of all five genes were significantly upregulated in the IAA group compared with those in the IAACon group, which exhibited correlation at the mRNA and protein abundance patterns (Figure 7). These results are consistent with those in Figure 1, that the expression of the proteins for IAA degradation is induced by IAA.

#### Identification of the Degradation Intermediates

The degradation pathway could provide the information about reaction type of each enzymatic catalyzing step, which could help to investigate the genes responsible for these reactions. Resting cell reactions were performed to identify substrates that could be transformed by strain LY1. Strain LY1 could transform IAA, catechol, 2-aminobenzoate, and isatin, while it could not transform indole, indolecarboxylic acid, indolebutyric acid, and indolepropionic acid. Furthermore, several rounds of resting cell reactions were performed to catch the IAA degradation intermediates, and the samples were analyzed by UV spectroscopy, HPLC, LC-MS, and GC-MS.

In resting cell reactions of Supplementary Figure 3, IAA was degraded and a new product (M#1) was accumulated. This product (M#1) was purified from the transformation products giving characteristic absorption peaks at 209, 254, and 291 nm (Figure 5). The *m*/*z* of product M#1 in LC-MS analysis is 206.0456, representing a molecular formula of C_10_H_9_NO_4_. NMR results confirmed that this compound was DOAA (Supplementary Figure 4). Product M#1 was putatively annotated as dioxindole-3-acetic acid (DOAA), a product of the initial few steps of IAA degradation. A degradation product (M#2) with characteristic absorption peaks of 226 and 362 was observed in the resting cells reaction (Supplementary Figure 5). M#2 was collected and analyzed by LC-MS. Product M#2 was detected with an *m*/*z* of 164.0356 (Figure 6), representing a molecular formula of C_8_H_7_NO_3_. IAA-grown LY1 cells could degrade isatin, and M#2 was also detected when isatin was used as the substrate in the resting cells reaction (Supplementary Figure 6A). Therefore, M#2 was identified as the hydrolysis product of isatin, isatinic acid. In the resting cells reaction samples, a product (M#3) with an *m*/*z* of 146.0250 was also detected (Supplementary Figure 6B), representing a molecular formula of C_8_H_5_NO_2_. Considering that isatin can be degraded by strain LY1 and they have the same degradation product, therefore, M#3 was suggested as isatin. A new peak (M#4) with a retention time of 6.59 min was observed in the HPLC signal (Supplementary Figure 7). The spectrum of peak M#4 revealed characteristic absorption peaks at 217, 245, and 333 nm (Supplementary Figure 7). The retention time and spectrum of peak M#4 are identical to those of authentic anthranilate. To further identify this product, the peak was collected from HPLC and analyzed by GC-MS. This product gives an *m*/*z* 266 in GC-MS result (Supplementary Figure 7), which corresponds to the 2-TMS alkylated anthranilate. The results confirmed that anthranilate was the degradation product of IAA in strain LY1.

#### Characterization of Genes in *iad* Cluster

To examine the role of genes located in the *iad* cluster in IAA degradation, *iadA*, *iadB*, and *iadE* were disrupted by homologous recombination to produce the mutants *Pseudomonas* sp. LY1Δ*iadA*, LY1Δ*iadB*, and LY1Δ*iadE*. The three mutants were unable to grow with IAA as the sole source of carbon and nitrogen. The results indicated that the three genes were essential for IAA degradation in strain LY1. The results were consistent with the proteomic analysis results. However, all three mutants could transform IAA, indicating that LY1 could co-transform IAA by other un-specifically expressed enzymes.

## Discussion

Indole-3-acetic acid degradation and cellular responses to IAA degradation in *Pseudomonas* sp. LY1 were studied here, revealing complex IAA degradation pathways and systematic response to this metabolic process. Several IAA degradation intermediates were detected in resting cell reactions including DOAA, isatin, isatinic acid, and anthranilate. DOAA is a degradation intermediate of the initial few steps in IAA degradation, which was reported in *B. japonicum* (Jensen et al., 1995), *Caballeronia glathei* DSM50014 (Sadauskas et al., 2020), and *P. putida* 1290 (Scott et al., 2013). In addition, IacA in strain 1290 and DSM50014 was demonstrated to be the candidate for the initial attack on IAA (Leveau and Gerards, 2008) to form 2-hydroxyindole-3-acetic acid. Therefore, 2-hydroxyindole-3-acetic acid was also suggested as one of the undetected intermediates in IAA degradation here. 2-Hydroxyindole-3-acetic acid was transformed to DOAA, which was then transformed to isatin, isatinic acid, and anthranilate as reported in *B. japonicum* (Jensen et al., 1995). Anthranilate and catechol are two major end products in microbial degradation of IAA (Arora et al., 2015; Laird et al., 2020). Although anthranilate can be transformed into catechol by anthranilate 1,2-dioxygenase [1.14.12.1] in indole degradation, anthranilate and catechol were not reported at the same IAA degradation pathway (Arora et al., 2015; Qu et al., 2017; Laird et al., 2020). In *P. putida* 1290, the IAA degradation pathway features catechol as a central metabolite. Catechol 1,2-dioxygenase activity increased in IAA-grown cells (Leveau and Lindow, 2005), and *cat* and *pca* genes were found to be essential to IAA degradation. The transformation from IAA to catechol has been proved heterologously in *E. coli* cells (Scott et al., 2013) through the change of the colony color and GC-MS analysis. The *iac* gene cluster was considered to be responsible for the catechol pathway of IAA degradation. No anthranilate was detected during IAA degradation in strains with the catechol pathway. In *E. soli* LF7, the expression of the *iac* gene cluster and catechol degradation genes were induced by IAA according to transcriptomics analysis. Although the degradation products were not identified experimentally, catechol was considered as the end product of the IAA degradation pathway in strain LF7 (Greenhut et al., 2018). In this study, strain LY1 contains all the homologous IAA degradation genes in strain 1290. Catechol was not detected in IAA degradation in strain LY1; however, the proteomic analysis proved that catechol or its derivatives exist in IAA degradation. Based on the genomic and proteomic analysis, a gene cluster, *iad* cluster, responsible for IAA degradation in strain LY1 was identified. Three gene sub-clusters were identified in the *iad* cluster, including *iadHABICDEFG* for IAA transformation, *catBCA* for catechol transformation, and *pcaAEFBDC* for protocatechuate transformation. Almost all the proteins in the *iad* cluster were significantly upregulated indicating that all three sub-clusters play a role in IAA degradation. In consideration of the catalytic reactions of *catBCA* and *pcaQEFBDC*, a phenolic compound with two *ortho*-hydroxy groups might be the main degradation intermediate in IAA degradation in strain LY1. Based on the experimental results, we speculated that IadCD was the key protein to break the pyrrole ring through an angular dioxygenation reaction to form phenolic intermediate with ortho-dihydroxy groups.

**FIGURE 8:**
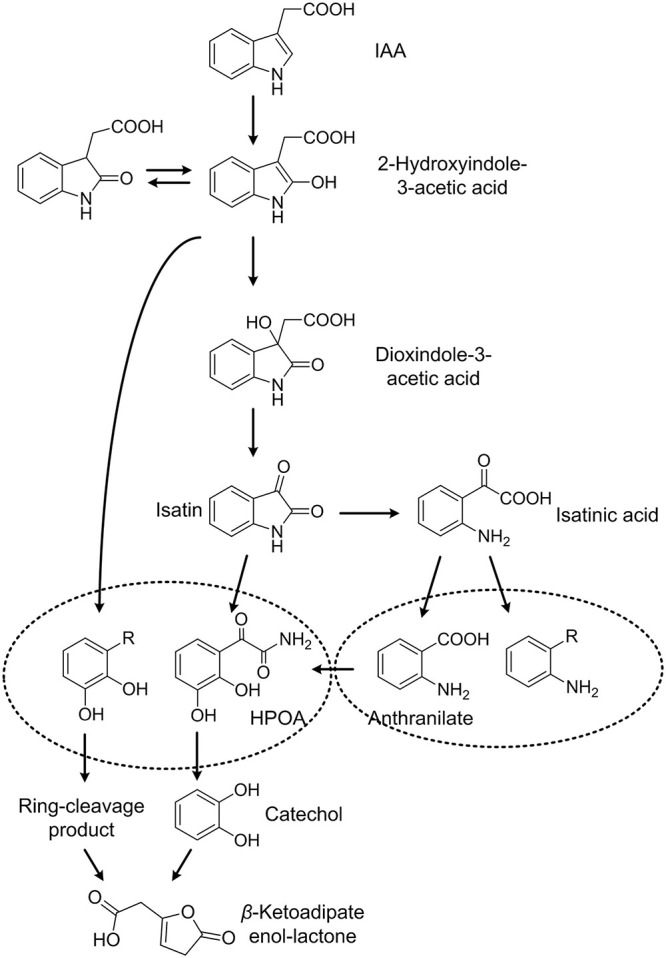
Proposed IAA degradation in strain *Pseudomonas* sp. LY1.

Isatin, isatinic acid, and anthranilate were demonstrated to be products of the anthranilate pathway of IAA degradation in *B. japonicum* USDA 110, and they were not reported in strains harboring *iac* gene clusters. Intriguingly, no *iac* genes were found in the genome of *B. japonicum* USDA 110 (Kaneko et al., 2002), which indicated that genes for the anthranilate pathway are different from that of the catechol pathway. Strain LY1 may contain other genes, rather than *iad* genes, that are responsible for the anthranilate pathway. In addition, although *E. coli* BL21, *E. coli* DH5ɑ, *E. coli* Trans1-T1, and *P. putida* KT2440 could not grow with IAA as the sole carbon and nitrogen source, all of these strains could completely transform IAA with a lower rate. The mutants we constructed in this study could not grow with IAA, but all the mutants could completely transform IAA. These results suggested that unspecific IAA degradation pathways exist in many bacteria as well as strain LY1. Therefore, multiple degradation pathways exist in strain LY1. Based on the experiment’s results and analysis, a tentative pathway for IAA degradation in *Pseudomonas* sp. LY1 is presented in Figure 8.

Proteomic analysis is a useful tool to elucidate the global protein response to pollutant degradation (Armengaud, 2016; Lyratzakis et al., 2021). As shown in Figure 5, IAA-up DAPs were mainly grouped into three biological processes: IAA degradation, stress response, and membrane-related processes, and the three biological processes have interactions with each other. Several proteins that were upregulated were associated with responses to stress response. In strain LY1, a UspA (PCLY0513) was upregulated in the IAA group. UspA is a small protein produced under stress, which acts as a defense involved in oxidative stress, and enhances the survival rate of cells. UspA abundance was significantly upregulated when *Arthrobacter* cells were induced by phenol (Li et al., 2016). The results indicated that LY1 cells indeed experienced a stressful situation during IAA degradation (Supplementary Table 3). Reaction oxygen species (ROS) are typical biomarkers in response to environmental stress in all organisms (Imlay, 2015; Bellenberg et al., 2019). ROS accumulation has been reported in the degradation of many pollutants, such as PHA, 17β-estradiol, and phenol (Li et al., 2012; Imlay, 2015). As reported, the degradation of aromatic compounds by mono-dioxygenases can generate ROS (Tamburro et al., 2004). The main antioxidant defense system is composed of antioxidant enzymes including catalase (CAT), superoxide dismutase (SOD), and peroxidase (POX). These enzymes could transform superoxide anions and hydrogen peroxide to water (Imlay, 2015). In strain LY1, two CATs (PCLY1673 and PCLY2596), two SODs (PCLY0007 and PCLY4730), and five POXs (PCLY0280, PCLY1828, PCLY1444, PCLY2878, and PCLY4135) were predicted, among which two were significantly upregulated in the IAA group and none was downregulated. The upregulation of these enzymes suggested that LY1 experienced ROS stress during IAA degradation. Glutathione is the most abundant antioxidant molecule in cells, and it protects against oxidative stress via direct interaction with ROS leading to glutathione disulfide or as the substrate of glutathione peroxidase (Tamburro et al., 2004; Imlay, 2015; Wongsaroj et al., 2018). Riccillo et al. (2000) reported that glutathione is important for acid tolerance, and osmotic and oxidative stresses in a *Rhizobium tropici* strain. Here, a glutamate cysteine ligase (PCLY0723) and γ-glutamyltranspeptidase (PCLY0482) were upregulated. The two enzymes are key enzymes in glutathione biosynthesis and both play roles in stress response. In *Chlamydomonas* sp. ICD-L, a glutamate cysteine ligase was upregulated in response to temperature and salinity stress (Peng et al., 2018). In humans, glutamate cysteine ligase and γ-glutamyltranspeptidase were upregulated in response to oxidative stress (Nicolae et al., 2017). Therefore, glutathione metabolism was involved in the defense of oxidative stress caused by IAA degradation in strain LY1.

Reaction oxygen species can damage the structure and activity of proteins, the folding of the protein, or the structure of the nucleic acids. Chaperones could help protein folding correctly, or binding to the activated protein in case of aggregation. Interestingly, the predicted heat shock proteins were not upregulated in the IAA group, but all the five predicted cold shock proteins (PCLY4010, PCLY3411, PCLY1477, PCLY3037, and PCLY4247) were upregulated from 1.36- to 2.49-fold. Cold shock protein could help to prevent RNA secondary structure formation. Sulfur-containing amino acids cysteine and methionine are particularly susceptible to ROS (Ezraty et al., 2017) and bacteria produce repair systems to protect proteins. Five enzymes [PCLY3111 (DsbC), PCLY4514, PCLY2272, PCLY1351, and PCLY0497] are involved in reducing system for repairing oxidatively damaged cysteine and methionine residues were predicted in LY1, and all of these enzymes were upregulated from 1.28- to 1.69-fold (Supplementary Table 3). IAA degradation has more influence on RNA structure, and cysteine and methionine residues of proteins, and has little influence on protein aggregation or folding.

Figure 4A shows that five of the six largest GO/CC terms of IAA-up DAPs are related to the membrane or periplasmic space, suggesting that membrane proteins played multiple roles in the cellular adaptation of IAA degradation in strain LY1. Transporter could directly affect the degradation capacity of bacteria. Xu et al. (2012) reported an active gentisate transporter, GenK, in *Corynebacterium glutamicum* ATCC13032, which was shown to be a limiting step for gentisate utilization. Expression of GenK could confer on *Ralstonia* sp. strain U2, which could not grow with gentisate, the ability to utilize gentisate. The transportation of IAA and its degradation intermediates might require specific transporters. An outer membrane porin (PCLY2669) was predicted in the *iad* cluster, and it showed the highest fold change (216.75) of all IAA-up DAPs, suggesting that this porin channel involved in the transportation of IAA or its degradation intermediates. KEGG enrichment showed five proteins, including the genes of PCLY2791, PCLY2792, PCLY2793, and PCLY2794, involved in β-lactam resistance (Figure 4B). Several γ-lactam intermediates in IAA degradation were detected; therefore, this gene cluster may be involved in the transportation of γ-lactam intermediates in strain LY1.

As oxidative stress is known to be present predominantly close to the cytoplasmic membrane, bacteria defend this stress by precisely adjusting their membrane lipid composition and expressing specific transporters (Zhang and Rock, 2008; Lushchak, 2011; Li et al., 2018). Three enzymes (PCLY0161, PCLY1283, and PCLY1410) involved in fatty acid biosynthesis and polyunsaturated fatty acid biosynthesis were significantly upregulated or expressed from undetectable to detectable in the IAA group. PCLY2859, 3-ketoacyl-CoA thiolase, was upregulated to 1.53-fold in the IAA group. Upregulation of these enzymes indicated that IAA degradation affected the membrane fluidity, and cells might defend against this by changing the structure or increasing the ratio of unsaturated polyunsaturated fatty acids.

Sixteen proteins involved in the two-component system, five in quorum sensing, were enriched in KEGG pathways (Figure 4B), and several transcriptional regulators were significantly upregulated. These proteins representing the degradation or stress sensing and regulation systems. Further investigation is required for characterizing the functions of these enzymes. A predicted chemoreceptor (PCLY2651) was upregulated by 16.1-fold, a chemotaxis protein A (PCLY2001) change from undetectable to detectable in the IAA group, and five proteins related to flagellar assembly were also significantly upregulated (Figure 4B) (Wadhams and Armitage, 2004). This suggested that strain LY1 could sense IAA or its degradation intermediates and might activate movement toward the substrate. This could increase the viability of strain LY1 in the environment with these compounds.

**FIGURE 9:**
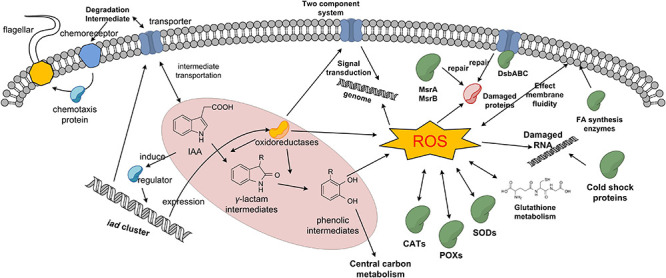
Proposed protein profile of IAA degradation response in strain LY1.

No central enzymes involved in carbon metabolism, amino acid biosynthesis, and oxidative phosphorylation had significant differential abundance. These results indicated that the core carbon and energy metabolism systems are relatively stable during IAA degradation. In summary, the proposed protein profile of IAA degradation response in strain LY1 is shown in Figure 9.

## Data Availability Statement

The datasets generated for this study can be found in online repositories. The names of the repository/repositories and accession number(s) can be found in the article/Supplementary Material.

## Author Contributions

SZ, HY, and XC conceived and designed the project. SZ, QS, FW, and CH performed the experiments. JB, HY, LG, and CH contributed reagents and materials. SZ, QS, HY, and XC analyzed the data. XC, HY, and CH wrote the article. All authors have read and approved the article.

## Conflict of Interest

The authors declare that the research was conducted in the absence of any commercial or financial relationships that could be construed as a potential conflict of interest.

## Publisher’s Note

All claims expressed in this article are solely those of the authors and do not necessarily represent those of their affiliated organizations, or those of the publisher, the editors and the reviewers. Any product that may be evaluated in this article, or claim that may be made by its manufacturer, is not guaranteed or endorsed by the publisher.
