# Supplementary material for: Label-Free Quantitative Proteomic Analysis of the Global Response to Indole-3-Acetic Acid in Newly Isolated Pseudomonas sp. Strain LY1
Source: Front Microbiol. 2021 Aug 10;12:694874. doi: 10.3389/fmicb.2021.694874 (PMC8383072; doi:10.3389/fmicb.2021.694874)
Supplement: Supplementary file 1 [file Table_1.docx]

**Label-free** **Quantitative Proteomic Analysis of the Global Response to Indole-3-acetic Acid in Newly Isolated *Pseudomonas* sp. strain LY1**

Shuxue Zhao^1,2^**^†^**, Xi Chen^1^**^†^**, Qianshu Sun^1,2^, Fei Wang^2^, Chunhui Hu^2^, Lizhong Guo^2^, Jie Bai^1^, Hao Yu^2*^

^1^College of Environmental Science and Engineering, Ocean University of China, Qingdao 266100, China; ^2^Shandong Provincial Key Laboratory of Applied Mycology, School of Life Sciences, Qingdao Agricultural University, 700 Changcheng Road, Chengyang District, Qingdao 266109, Shandong Province, People's Republic of China

Key words: proteomics, microbial degradation, indole-3-acetic acid, *Pseudomonas* sp. LY1

*To whom correspondence should be addressed:

Hao Yu: yuhaosunshine@163.com

†Shuxue Zhao and Xi Chen contributed equally to this work.

**Supplementary Table 1 Primers used in this study.**

| Primer Name | Sequence (5′–3′) | Function Description (可选) |
| --- | --- | --- |
| *iadA*-RT-F | GCCCCTGGAGACGCTGGACAA | Used to quantify the expression level of *iadA* genes in RT-qPCR reactions |
| *iadA*-RT-R | TTTCCCGTGGCATCACGGCC |  |
| *iadB*-RT-F | CCCACGTGAGGAGCAGCTGG | Used to quantify the expression level of *iadB* genes in RT-qPCR reactions |
| *iadB*-RT-R | GGTGGTGCGGAACAGGTTGC |  |
| *iadC*-RT-F | GATCTGGAAGCCGACCTGGA | Used to quantify the expression level of *iadC* genes in RT-qPCR reactions |
| *iadC*-RT-R | CGCGTGGTGGATCTCGGTCT |  |
| *iadD*-RT-F | GAGACCGATTTCGAGAACAC | Used to quantify the expression level of *iadD* genes in RT-qPCR reactions |
| *iadD*-RT-R | CTTGAGCACGTCCTTGCGGA |  |
| *iadE*-RT-F | GCTGCGATGACCCGCACCAC | Used to quantify the expression level of *iadE* genes in RT-qPCR reactions |
| *iadE*-RT-R | CGATGGCGCCCTTGGAGGCC |  |
| 16S-RT-F | GGTAGTCCACGCCGTAAACGA | Used to quantify the expression level of 16S rRNA gene genes in RT-qPCR reactions |
| 16S-RT-R | CCAATCCATCTCTGGAAAGT |  |
| *iadA*-Mu-UF | TATGACATGATTACGAATTAGGCGGCGGATTTCGCACAG | Used for the construction of double crossover homologous recombination plasmid pK18MST-Δ*iadA* |
| *iadA*-Mu-UR | CGCTCACCACGCCGTTGGCGTACACCTT |  |
| *iadA*-Mu-DF | CGCCAACGGCGTGGTGAGCGTCGAGCAG |  |
| *iadA*-Mu-DR | CGGGTACCGAGCTCGAATTTGGTCATCCTCGACACGG |  |
| *iadB*-Mu-UF | TATGACATGATTACGAATTATTCTGCGAGATGGTGGAGG | Used for the construction of double crossover homologous recombination plasmid pK18MST-Δ*iadB* |
| *iadB*-Mu-UR | GTCTCGATGTCCGGCACGTCGGCCACCGCCCAGACTTCCAGCTGC |  |
| *iadB*-Mu-DF | AGCAGCTGGAAGTCTGGGCGGTGGCCGACGTGCCGGACATC |  |
| *iadB*-Mu-DR | AGGATCCCCGGGTACCGAGCTCGTCGCCCTTGTCCAGGCAGTC |  |
| *iadE*-Mu-UF | TATGACATGATTACGAATTTCCACCACGCGGTGATCA | Used for the construction of double crossover homologous recombination plasmid pK18MST-Δ*iadE* |
| *iadE*-Mu-UR | GGGGGCGATGGCGTTGACGATGGACAGACCGAGGC |  |
| *iadE*-Mu-DF | GCCTCGGTCTGTCCATCGTCAACGCCATCGCCCCC |  |
| *iadE*-Mu-DR | CGGGTACCGAGCTCGAATTTGCGTATCGCCTTCGTCAT |  |

**Supplementary Figure 1**

**
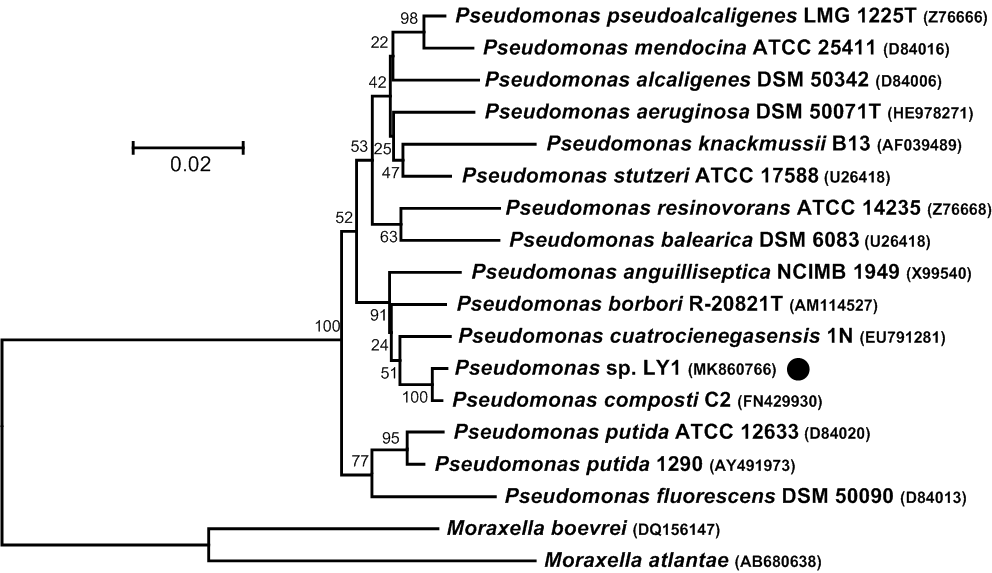
**

**Neighbour-joining tree based on analysis of the 16S rRNA gene showing relationship between strain LY1 and relative *Pseudomonas* sp. strains.** 16S rRNA genes from *Moraxella* were used as outgroup. Bootstrap probabilities (as percentages) are determined from 1,000 resamplings. Bar 0.005 substitutions per nucleotide position.

**Supplementary Figure 2**

**
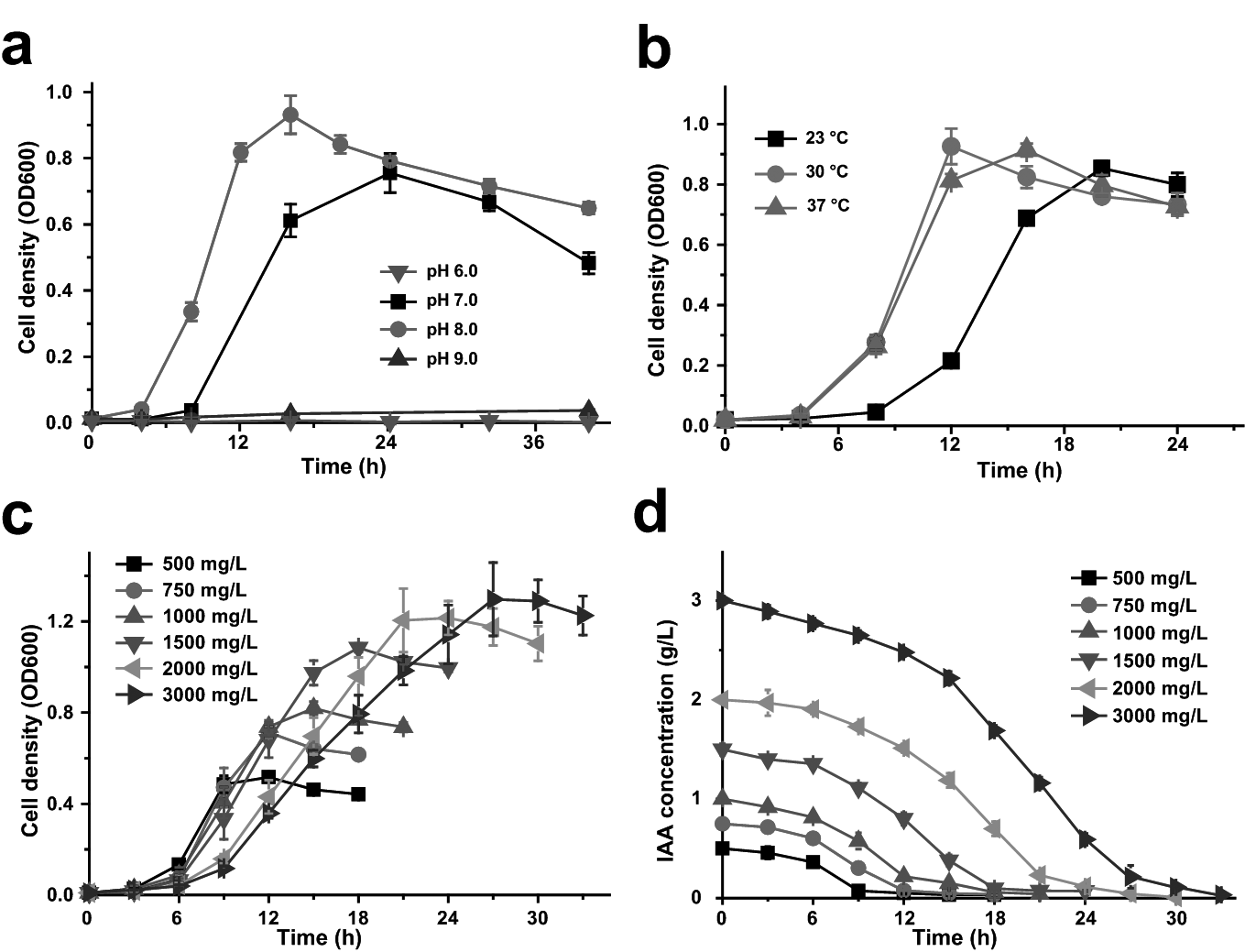
**

**Growth of strain LY1 under different conditions.** a. Effect of pH on the growth of strain LY1; b. Effect of temperature on the growth of strain LY1. c. Effect of IAA concentration on the growth of strain LY1. d. The changes of IAA concentrations in LY1 cultures. Each value is the mean from three parallel replicates ± SD.

**Supplementary Figure 3**


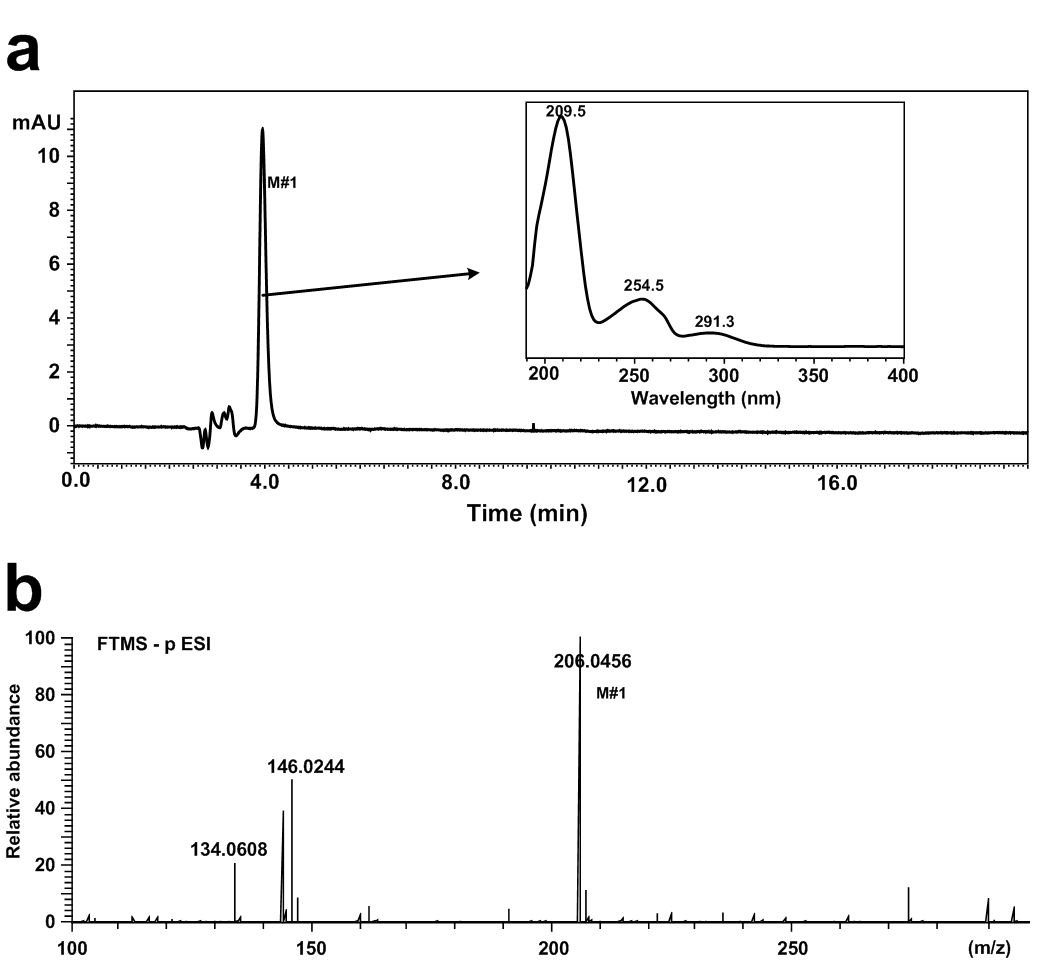


**Identification of dioxindole-3-acetic acid as the IAA degradation intermediate.** a. HPLC analysis of the collected new intermediate. b. LC-MS analysis of the new intermediate.

**Supplementary Figure 4**


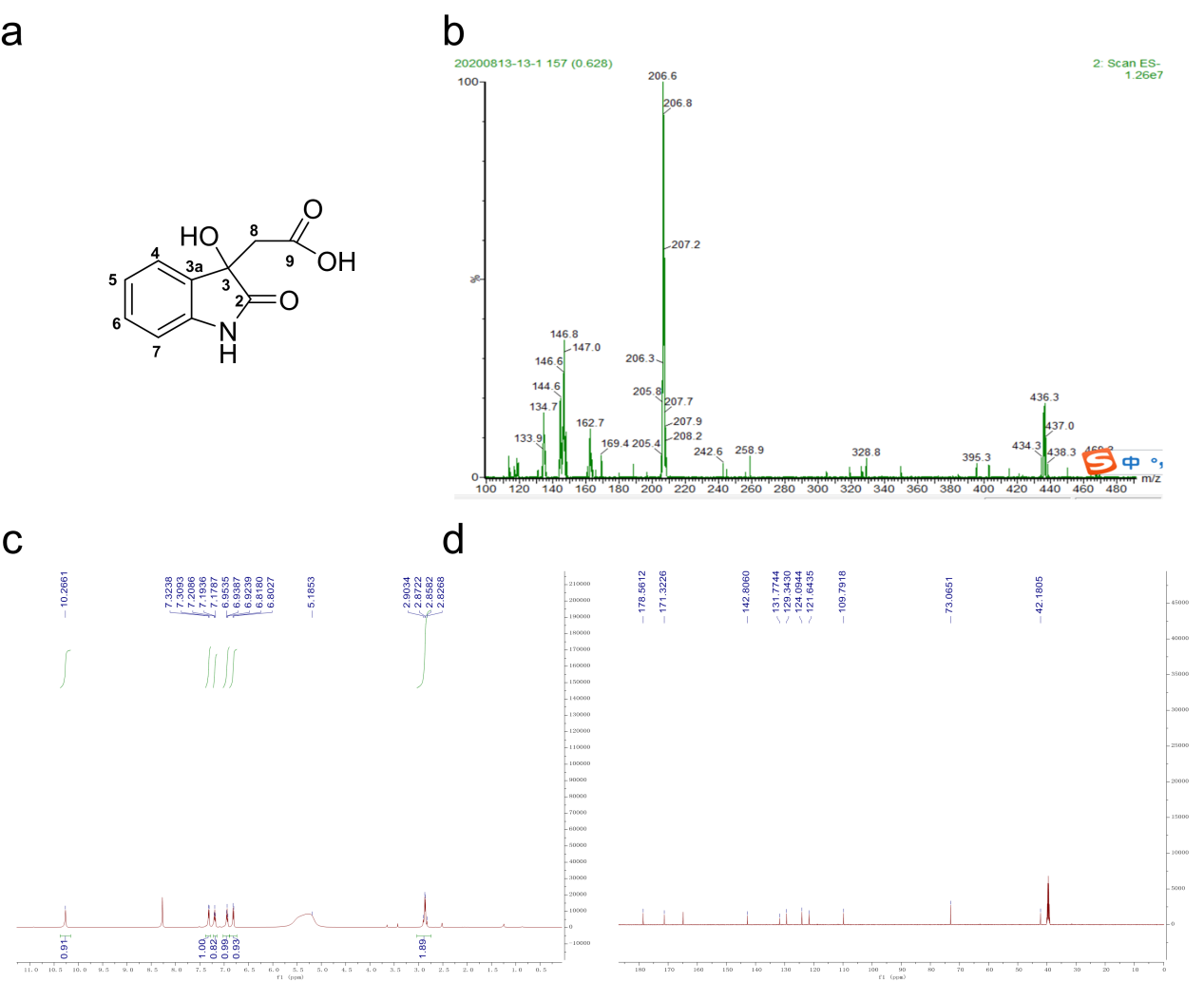


RNM results. (a) structure of **M#1**. (b) ESIMS spectrum of **M#1**; (c) ^1^H-NMR spectrum of (**1**) in DMSO-*d*_6_ (d) ^13^C-NMR spectrum of **M#1** in DMSO-*d*_6_. Compound **M#1** was obtained as white amorphous powder. The molecular formula was C_10_H_9_NO_4_ on the basis of a ESIMS peak at m/z 206.6[M-H]^-^ and NMR data, indicating 7 degrees of unsaturation. The observed signals in ^1^H NMR spectrum of **M#1** for two doublet aromatic protons at δ_H_ 7.31 (d, J = 7.3) and 6.81 (d, J = 7.6), and two triplet aromatic protons at δ_H_ 6.94 (t, J = 7.4) and 7.19 (t, J = 7.5) were indicative of a 1,2-disubstituted benzene ring system . The ^13^C NMR spectra revealed the presence of 10 carbon atoms, which were clarified into five non-protonated carbons (including one carboxyl, one amide carbonyl, two olefinic and one oxygenated sp^3^ carbons), four olefinic methines and one methylenes. Thus, compound **M#1** was identified as 2,3-dihydro-3-hydroxy-2-oxo-1H-indole-3-acetic acid.

| NMR data of compounds **1** in DMSO-*d*_6_ (500 MHz for ^1^H and 125 MHz for ^13^C, *δ* in ppm, *J* in Hz) | | |
| --- | --- | --- |
|  | *δ*c | *δ*_H_ |
| 2 | 178.6, C |  |
| 3 | 73.1, C |  |
| 3a | 131.8, C | - |
| 4 | 124.1, CH | 7.31, d (7.3) |
| 5 | 121.6 CH | 6.94, t (7.4,) |
| 6 | 129.3, CH | 7.19, t (7.5) |
| 7 | 109.8, CH | 6.81,d (7.6) |
| 7a | 142.8, C | - |
| 8 | 42.2, CH_2_ | 2.90, d (15.6);  2.83, d (15.6) |
| 9 | 171.3, C |  |
| 1-NH |  | 10.27, s |

**Supplementary Figure 5**


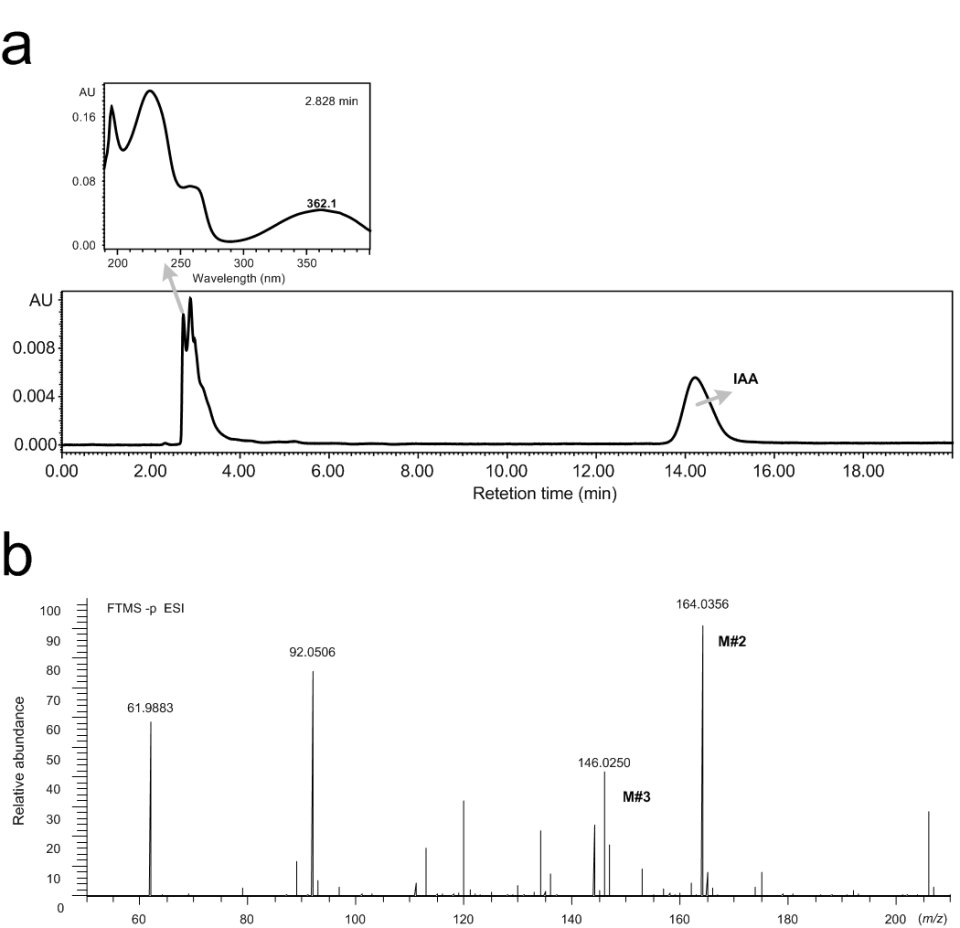


**Identification of isatin and 2-aminophenyl glyoxylic acid as the IAA degradation intermediate.** a. HPLC analysis of the collected new intermediate. b. LC-MS analysis of the new intermediate.

**Supplementary Figure 6**


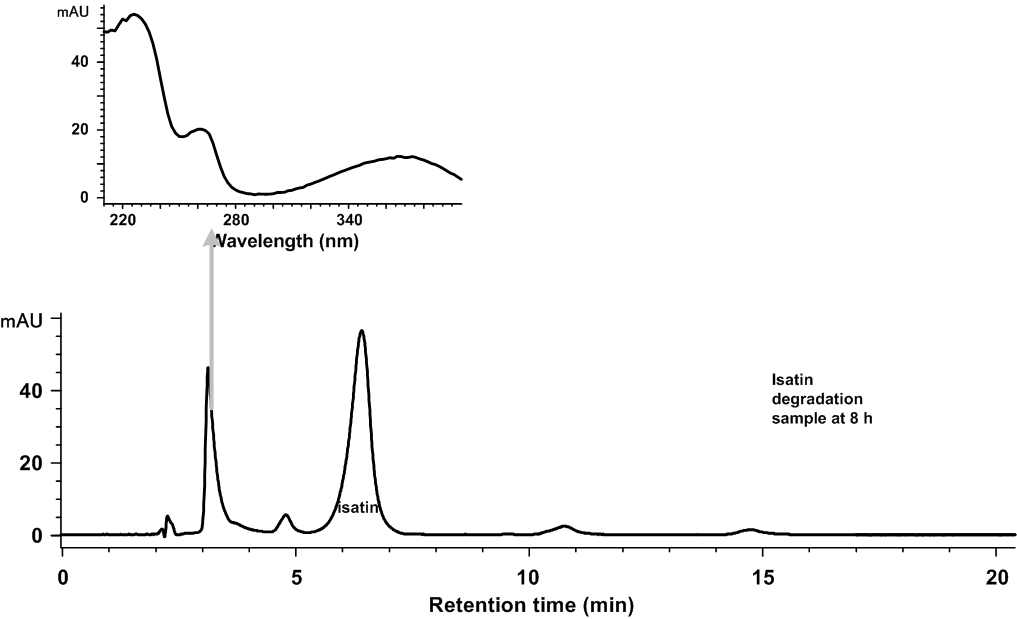


**Isatin transformation by IAA induced resting cells of strain LY1.** The signal at 254 nm was recorded. Isatin (with a retention time of 6.4 min) was transformed to a new compound (M#2, isatinic acid) with a retention time of 3.1 min. The arrow indicated UV spectrum of M#2. The mobile phase was 35% (v/v) methanol and 65% (v/v) 0.05% formic acid. The HPLC column was Agilent XBD C18 (4.6 mm × 250 mm, 5 μm).

**Supplementary Figure 7**


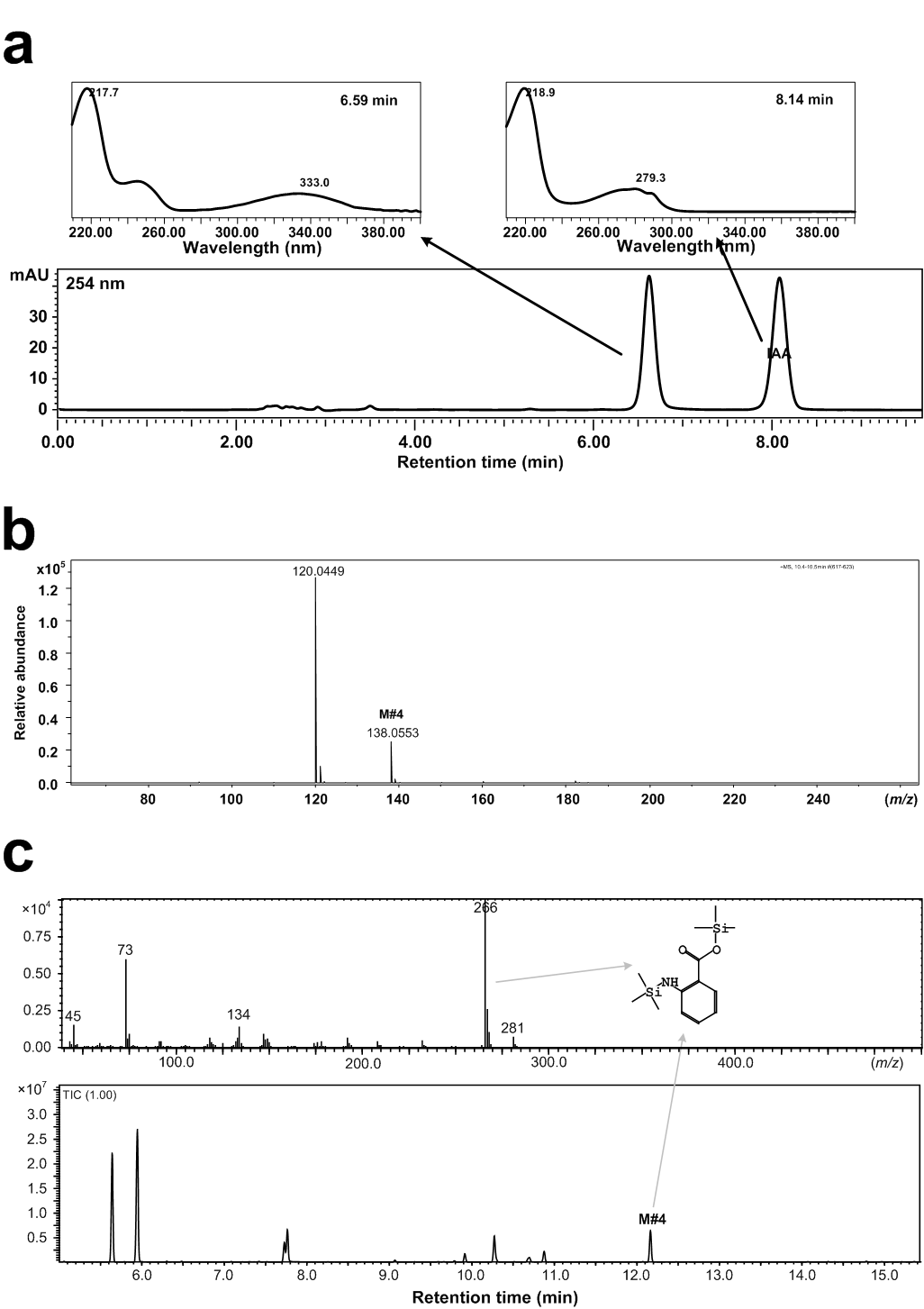


**Identification of anthranilate as the IAA degradation intermediate by strain LY1.** a. HPLC analysis of the sample of IAA transformation by IAA-grown resting cells of strain LY1. Spectra of the instinct peaks from HPLC DAD detector were also shown. b. LC-MS analysis of the purified compound with the retention time of 6.59 min in HPLC analysis (a). c. GC-MS analysis of the purified compound.
